# Systemic LRG1 Expression in Melanoma is Associated with Disease Progression and Recurrence

**DOI:** 10.1158/2767-9764.CRC-23-0015

**Published:** 2023-04-20

**Authors:** Esmee P. Hoefsmit, Franziska Völlmy, Elisa A. Rozeman, Irene L.M. Reijers, Judith M. Versluis, Liesbeth Hoekman, Alexander C.J. van Akkooi, Georgina V. Long, Dirk Schadendorf, Reinhard Dummer, Maarten Altelaar, Christian U. Blank

**Affiliations:** 1 Department of Molecular Oncology and Immunology, Netherlands Cancer Institute, Amsterdam, the Netherlands.; 2 Biomolecular Mass Spectrometry and Proteomics, Center for Biomolecular Research and Utrecht Institute for Pharmaceutical Sciences, Utrecht University, Utrecht, the Netherlands.; 3 Department of Medical Oncology, Netherlands Cancer Institute, Amsterdam, the Netherlands.; 4 Proteomics Facility, Netherlands Cancer Institute, Amsterdam, the Netherlands.; 5 Melanoma Institute Australia, The University of Sydney, Sydney, New South Wales, Australia.; 6 Faculty of Medicine and Health, The University of Sydney, Sydney, New South Wales, Australia.; 7 Department of Melanoma and Surgical Oncology, Royal Prince Alfred Hospital, Sydney, New South Wales, Australia.; 8 Charles Perkins Centre, The University of Sydney, Sydney, New South Wales, Australia.; 9 Department of Medical Oncology, Royal North Shore and Mater Hospitals, Sydney, New South Wales, Australia.; 10 Department of Dermatology, University Hospital Essen and Germany Cancer Consortium, Partner Site Essen, Essen, Germany.; 11 Department of Dermatology, University Hospital Zürich, University Zürich, Zürich, Switzerland.; 12 Department of Medical Oncology, Leiden University Medical Center, Leiden, the Netherlands.

## Abstract

The response rates upon neoadjuvant immune checkpoint blockade (ICB) in stage III melanoma are higher as compared with stage IV disease. Given that successful ICB depends on systemic immune response, we hypothesized that systemic immune suppression might be a mechanism responsible for lower response rates in late-stage disease, and also potentially with disease recurrence in early-stage disease. Plasma and serum samples of cohorts of patients with melanoma were analyzed for circulating proteins using mass spectrometry proteomic profiling and Olink proteomic assay. A cohort of paired samples of patients with stage III that progressed to stage IV disease (*n* = 64) was used to identify markers associated with higher tumor burden. Baseline patient samples from the OpACIN-neo study (*n* = 83) and PRADO study (*n* = 49; NCT02977052) were used as two independent cohorts to analyze whether the potential identified markers are also associated with disease recurrence after neoadjuvant ICB therapy. When comparing baseline proteins overlapping between patients with progressive disease and patients with recurrent disease, we found leucine-rich alpha-2-glycoprotein 1 (LRG1) to be associated with worse prognosis. Especially nonresponder patients to neoadjuvant ICB (OpACIN-neo) with high LRG1 expression had a poor outcome with an estimated 36-month event-free survival of 14% as compared with 83% for nonresponders with a low LRG1 expression (*P* = 0.014). This finding was validated in an independent cohort (*P* = 0.0021). LRG1 can be used as a biomarker to identify patients with high risk for disease progression and recurrence, and might be a target to be combined with neoadjuvant ICB.

**Significance:**

LRG1 could serve as a potential target and as a biomarker to identify patients with high risk for disease recurrence, and consequently benefit from additional therapies and intensive follow-up.

## Introduction

Immune checkpoint blockade (ICB) using anti-programmed cell death 1 (anti-PD-1) antibodies, either as monotherapy, or in combination with anti-CTL-associated protein 4 (anti-CTLA-4) antibodies, is currently one of the most effective standard therapies for late-stage melanoma (1–5). In cross-trial comparison, a higher response rate to ICB is observed for patients with stage III as compared with stage IV disease (5–8). In addition, high-grade (grade 3–4) immunotherapy-related adverse events (irAE) are more frequently observed in stage III melanoma than would be predicted from prior data in stage IV melanoma at similar dosing of ipilimumab 3 mg kg^−1^ plus nivolumab 1 mg kg^−1^ and number of courses (90% in stage III vs. 59% in stage IV; refs. 5, 6). The lower response rate and the lower irAE rates suggest that patients with late-stage melanoma may have a higher level of systemic tumor-associated immune suppression hampering ICB therapy (9).

The theory of systemic immune suppression is supported by analyses of immunocompetence showing that the proliferative capacity of peripheral lymphocytes decreases with disease progression (10). Given that successful ICB is reliant upon a systemic immune response (9), additional probing of this immune modulation could be beneficial to improve our understanding of the underlying lower response rate to ICB therapy in more advanced disease. Previous systemic proteomic biomarker analysis to distinguish early- and late-stage patients already identified few markers, including S100B, lactate dehydrogenase (LDH), C-reactive protein (CRP), and serum amyloid A (SAA; refs. 11–15). A remaining challenge is to identify low-level abundant protein biomarkers for disease progression and recurrence.

This prompted us to analyze a cohort of patients with melanoma for circulating proteins using mass spectrometry (MS)-based protein profiling in an unbiased approach (16, 17). In this study, we analyzed serum of patients with melanoma to identify systemic biomarkers that are associated with disease progression and recurrence in early-stage disease.

## Materials and Methods

### Study Population

**FIGURE 1:**
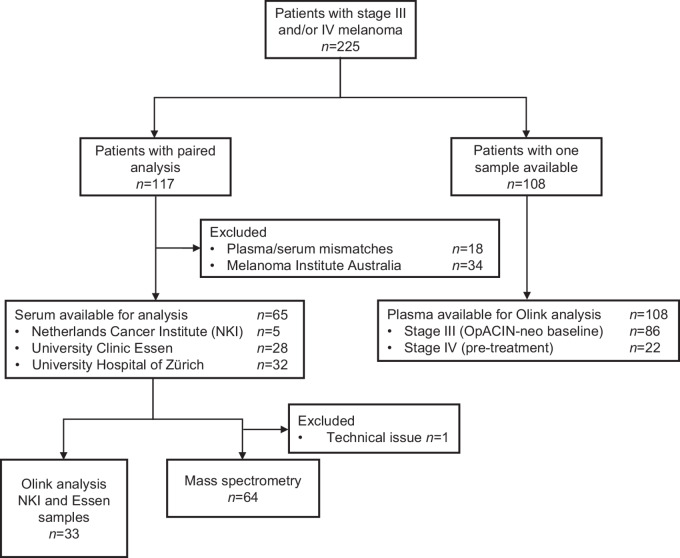
Flowchart of patients with stage III and stage IV melanoma selected for serum/plasma analyses. Number of patients included for the different analysis. A total of 117 patients with paired plasma/serum samples were available at stage III disease and stage IV disease (paired analysis). Because of plasma/serum mismatch and heterogeneity between samples from European centers and MIA, the sera of 65 patients were used for analysis. Of these patients, 33 patients were analyzed by Olink and 64 patients were analyzed by MS (left). Plasma of 108 patients with stage III or stage IV was analyzed by Olink (right).

Patients with stage III melanoma that progressed to stage IV melanoma, from whom plasma/serum samples were available at both timepoints and were systemic treatment naïve at collection, were identified from four different cancer centers [Netherlands Cancer Institute (NKI), University Clinic Essen, University Hospital of Zürich, and Melanoma Institute Australia (MIA)]. Because of plasma/serum mismatches (samples within a patient) and heterogeneity between samples from European centers and MIA, we finally analyzed a more homogeneous cohort of paired serum samples from patients from the NKI (*n* = 5), University Clinic Essen (*n* = 28), and University Hospital of Zürich (*n* = 32). For serum sampling, blood was collected, spun down immediately after isolation and serum was harvested, snap-frozen, and stored. These paired stage III melanoma and stage IV melanoma serum samples were used to determine difference in soluble factors by MS-based proteomic analysis (*n* = 64) and proximity extension assay (*n* = 33; Olink Bioscience AB; Fig. 1).

In another cohort of patients, only plasma samples were available for MS-based proteomic analysis. This cohort included patients treated with ICB to determine difference in soluble factors between patients with and without a disease recurrence after ICB treatment. Plasma was obtained from whole blood collected in ethylene diamine tetra acetic acid (EDTA) tubes and stored at −80°C after spinning. This cohort included baseline (pretreatment) samples (*n* = 83 patients) and posttreatment (week 6) samples (*n* = 83 patients) of the OpACIN-neo study (NCT02977052; Supplementary Fig. S1). Hematology (including white blood cell differentiation) was tested baseline (pretreatment). The OpACIN-neo study tested three different dosing schedules of neoadjuvant ipilimumab plus nivolumab in stage III melanoma (A: two cycles ipilimumab 3 mg kg^−1^ plus nivolumab 1 mg kg^−1^ every 3 weeks; B: two cycles ipilimumab 1 mg kg^−1^ plus nivolumab 3 mg kg^−1^ every 3 weeks; C: two cycles ipilimumab 3 mg kg^−1^ every 3 weeks directly followed by two cycles nivolumab 3 mg kg^−1^ every 2 weeks; refs. 7, 18).

To evaluate whether the findings of the proximity extension assay (Olink Bioscience AB) could be confirmed in an independent cohort, pretreatment plasma samples from patients with stage IV melanoma (*n* = 22 patients) were collected at the NKI and compared with pretreatment plasma samples from patients with stage III melanoma from the OpACIN-neo study (*n* = 86 patients; Fig. 1). The study was approved by the Institutional Review Board of the NKI.

For validation cohort, baseline (pretreatment) plasma samples of the PRADO study (*n* = 49) were selected (ref. 8; Supplementary Fig. S2). In this study, patients were also treated with neoadjuvant ipilimumab and nivolumab. The studies were conducted in accordance with Good Clinical Practice guidelines as defined by the International Conference of Harmonization and Declaration of Helsinki.

### Ethics Approval

All retrospective medical data/biospecimen studies at the NKI have been executed pursuant to Dutch legislation and international standards (reference CFMPB558, N03LAM, OpACIN-neo, PRADO). Prior to May 25, 2018, national legislation on data protection applied, as well as the International Guideline on Good Clinical Practice. From May 25, 2019, we also adhere to the GDPR. Within this framework, patients are informed and have always had the opportunely to object or actively consent to the (continued) use of their personal data and biospecimens in research. Hence the procedure comply both with (inter) national legislative and ethical standards. All University Hospital Essen and Germany Cancer Consortium patient samples included in this study were collected covered by EC vote BO-11-4715 (Essen) with written consent. All University Hospital Zürich patient samples have signed a release form, which have been approved by the ethics committee and assigned the numbers EK647 and EK800.

### Sample Preparation for Proteomic Analysis of Stage III and Stage IV Patients

For the serum samples, total protein concentration was determined using a Bradford assay and 600 μg worth of protein was loaded onto a Pierce Top 12 Abundant Protein Depletion Spin column (Thermo Fisher Scientific), followed by a 1-hour end-on-end rotating incubation at room temperature. The depleted serum was collected by centrifuging for 2 minutes at 1000G and each sample was split in two to generate parallel workflow duplicates. A detergent-based buffer [1% sodium deoxycholate (SDC), 10 mmol/L tris(2-carboxyethyl)phosphine (TCEP), 10 mmol/L Tris, 40 mmol/L chloroacetamide] with Complete mini EDTA-free protease inhibitor cocktail (Roche) was added to enhance protein denaturation and boiled for 5 minutes at 95°C. A total of 50 mmol/L ammonium bicarbonate was added and digestion was allowed to proceed overnight at 37°C using trypsin (Promega) and LysC (Wako) at 1:50 and 1:75 enzyme:substrate ratios, respectively. The digestion was quenched with 10% formic acid (FA) and the resulting peptides were cleaned up using Oasis HLB 96-well uElution plates (Waters Corporation). The eluate was dried and resolubilized in 1% FA achieving a concentration of 1 μg/μL. HRM iRT retention time peptides (Biognosys) were spiked in following the producer's recommendations and finally an on-column peptide load of 1.5 μg peptides was achieved.

### Sample Preparation for Proteomic Analysis of OpACIN-neo and PRADO Plasma

To enhance protein denaturation, 24 μL of a detergent-based buffer (1% SDC, 10 mmol/L TCEP, 10 mmol/L Tris, and 40 mmol/L chloroacetamide) with Complete mini EDTA-free protease inhibitor cocktail (Roche) was added to 1 μL plasma and boiled for 5 minutes at 95°C. A total of 50 mmol/L ammonium bicarbonate was added and digestion was allowed to proceed overnight at 37°C using trypsin (Promega) and LysC (Wako) at 1:50 and 1:75 enzyme:substrate ratios, respectively. For OpACIN-neo samples, the digestion was quenched with 10% formic acid and the resulting peptides were cleaned up in an automated fashion using the AssayMap Bravo platform (Agilent Technologies) with corresponding AssayMap C18 reverse-phase column. The eluate was dried and resolubilized in 1% FA to achieve a concentration of 1 μg/μL, of which 1 μL was injected. For PRADO samples, the digestion was quenched by the addition of trifluoroacetic acid (final concentration 1%), after which the peptides were desalted using C18 StageTips (Thermo Fisher Scientific). Samples were dried in a vacuum centrifuge and reconstituted in 2% formic acid for MS analysis.

### Data Independent Acquisition LC/MS-MS Analysis

All spectra were acquired on an Orbitrap HFX mass spectrometer (Thermo Fisher Scientific) for OpACIN-neo and stage III/IV cohort samples and Orbitrap Exploris 480 Mass Spectrometer with a FAIMS-PRO interface (Thermo Fisher Scientific) for PRADO samples, operated in data independent acquisition mode (DIA) coupled to an EASY-nLC 1200 liquid chromatography pump (Thermo Fisher Scientific) and separated on a 50 cm reversed phase column (Thermo Fisher Scientific, PepMap RSLC C18, 2 M, 100A, 75 m × 50 cm) for OpACIN-neo and stage III/IV cohort samples and on a 25 cm reversed phase column (Thermo Fisher Scientific, PepMap RSLC C18, 2 M, 100A, 75 μm × 25 cm) for PRADO samples.

For OpACIN-neo samples, proteome samples were eluted over a linear gradient ranging from 5% to 25% acetonitrile over 100 minutes, 25%–100% acetronitrile for 5 minutes, followed by 100% acetonitrile for the final 15 minutes with a flow rate of 200 nL/minute. DIA runs consisted of a MS1 scan at 60,000 resolution at m/z 200 followed by 30 sequential quadrupole isolation windows of 20 m/z for higher energy collision dissociation (HCD) MS-MS with detection of fragment ions in the orbitrap (OT) at 30,000 resolution at m/z 200. The m/z range covered was 400–1,200 and the Automatic Gain Control (AGC) was set to 1e6 for MS and 2e5 for MS-MS. The injection time was set to 100 ms for MS and “auto” for MS-MS scans.

For PRADO samples, proteome samples were eluted from the analytical column at a constant flow of 250 nL/minute in a 60-minute gradient, containing a 50-minute linear increase from 6% to 30% solvent B, followed by a 10-minute wash at 90% solvent B. FAIMS was operated in the standard resolution mode, with additional FAIMS gas flow of 3.5 L/minute. DIA runs consisted of a MS1 scan at 120,000 resolution at m/z 200 followed by 39 sequential quadrupole isolation windows of 15 m/z for HCD MS-MS with detection of fragment ions in the OT at 30,000 resolution at m/z 200. The m/z range covered was 400–1,000 and FAIMS CV was set to −45 V. The injection time was set to 45 ms for MS and “auto” for MS-MS scans.

### Data Dependent Acquisition LC/MS-MS Analysis for the Stage III/IV Cohort

A sample made of pooled representative patient serum was fractionated and fractions were injected on the same setup and gradient as the DIA experiment (as for OpACIN-neo), but the spectra were acquired in data dependent acquisition fashion (DDA) to survey proteome composition in depth. The DDA data were acquired using a top-12 method where MS1 spectra had a resolving power of 60,000 at 200 m/z with an AGC target of 3e6 ions and a maximum injection time of 20 ms. MS-MS spectra were acquired with HCD fragmentation, a normalized collision energy of 27, a 1.4-m/z-wide isolation window, a resolving power of 30,000 at 200 m/z, an AGC target of 1e5 ions and a maximum injection time of 50 ms.

### Multiplex Proteomics Profiling

Proteins in plasma and serum samples were profiled by a multiplex assay using proximity extension assay technology (Olink Bioscience AB). A first set of serum samples of paired patient samples (*n* = 33 patients) with stage III melanoma that progressed to stage IV melanoma were selected for investigation. To validate the findings from the first set, pretreatment plasma samples from a second cohort of patients with stage III melanoma (OpACIN-neo study, *n* = 86 patients) and treatment-naïve patients with stage IV melanoma (*n* = 22 patients) were selected for analysis. The Olink Immuno-Oncology panel was selected, which allows simultaneously measurement of 92 analytes by binding of oligonucleotide-labeled antibody probe pairs to their targeted protein. When in close proximity, the oligonucleotides of the probes will hybridize in a pairwise manner and can be detected and quantified using real-time PCR. The assay was performed at the Department of Clinical Chemistry and Hematology at the University Medical Center Utrecht. More information about the Immuno-Oncology panel, detection limits, data quantification, normalization and standardization are available on the manufacturer's website: https://www.olink.com/resources-support/document-download-center/. Analysis of the samples was performed in R (R Foundation for Statistical Computing).

### Data Handling and Statistical Analysis

For the stage III/IV cohort, the proteins identified from DDA files were submitted to the Prosit tool (19) whereby artificial spectra were predicted, effectively generating an artificial spectral library. This library was in turn used to extract spectra from the DIA data using DIA-NN (20). The DIA-NN settings were as follows: “Deep learning” was enabled. The enzyme for digestion was set to trypsin with one missed cleavage tolerated and C Carbamidomethylation and M oxidation were allowed as variable modification. The precursor FDR was set to 1%. Protein grouping was done by protein names and cross-run normalization was RT dependent. The gene-centric report was used for downstream analysis, and all runs where less than 300 proteins were identified were discarded. Technical workflow replicates were combined by taking the log_2_ mean value per protein. The same data handling was carried out for the OpACIN-neo study, however no spectral library was used as this was constructed directly within the DIA-NN software using the DIA raw data as basis.

For the PRADO study, the raw data reads were analyzed by DIA-NN (version 1.8; ref. 20) without a spectral library and with “Deep learning” option enabled. The Swissprot human database (20,375 entries, release 2022_02) fasta was added for the library-free search. The quantification strategy was set to Robust LC (high accuracy) and MBR option was enabled. The other settings were kept at the default values. The protein groups report from DIA-NN was used for downstream analysis in Perseus (version 1.6.15.0; ref. 21). Values were log_2_ transformed, after which proteins were filtered for at least 70% valid values in at least one sample group

For paired patient samples, a paired two-tailed Student *t* test was used to compare the mean log_2_ values. Two-tailed Student *t* test and the Welch *t* test were used to compare the proteins abundance means. Additional information about quantification and statistical analyses performed are described in the corresponding figure legends. *P* value lower than 0.05 was regarded statistically significant. *, *P* < 0.05; **, *P* <0.01; ***, *P* <0.001; ****, *P* <0.0001.

All graphic visuals and statistical analysis were performed using Prism (Graphpad Software Inc., version 9) or in R (version 4.0.4) and R studio (version 1.4.1106) using the packages survminer (version 0.4.9), ggplot2 (version 3.3.5), cutpointr (version 1.1.2), ROCit (version 2.1.1), and RColorBrewer (version1.1.-3).

### Data Availability Statement

Data are available upon reasonable request. The MS proteomic data have been deposited to the ProteomeXchange Consortium via the PRIDE partner repository (22) with the dataset identifier PXD04399.

## Results

### Previously Identified CRP, SAA1, LDHB, IL8, and IL10 are Associated with Melanoma Disease Progression

**FIGURE 2:**
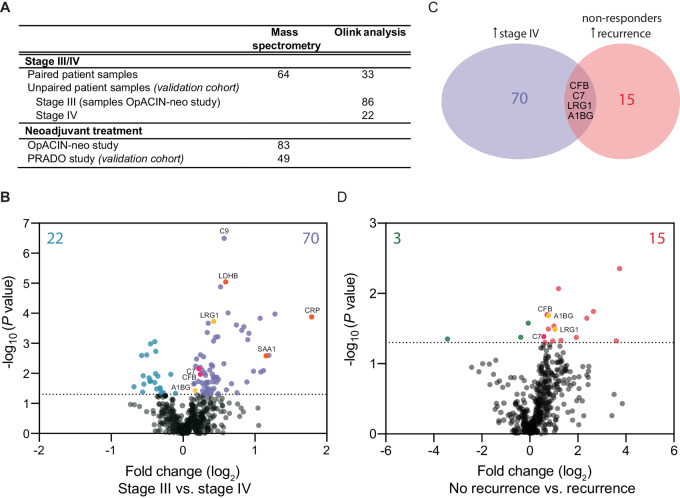
MS analysis of protein change upon disease progression and recurrence. **A,** Plasma and serum samples of different cohorts of patient with melanoma analyzed for circulating proteins using MS proteomic profiling and Olink analysis. **B,** Volcano plot showing differential protein expression of serum analysis using MS, comparing protein expression between matched patients with stage III and stage IV disease (*n* = 64). Proteins higher expressed at stage III disease are displayed on the left, and proteins higher expressed at stage IV are displayed on the right. The protein fold change on a log_2_ scale is shown on the *x*-axis, with the significance indicated by the −log_10_ scale on the *y*-axis. The significance cutoff (*P* = 0.05) is indicated with the black dotted line, showing significant increased proteins for stage III in blue and for stage IV in purple. A two-tailed paired Student *t* test was used to determine statistical significance between stage III and stage IV samples. **C,** A Venn diagram for overlapping significant proteins of patients with stage IV melanoma and baseline samples of nonresponding stage III patients with recurrent disease. **D,** Volcano plot showing differential expression of plasma markers using MS, comparing protein expression of nonresponder patients of the OpACIN-neo study (*n* = 21) with or without a recurrence. Proteins higher expressed by patients without a recurrence are displayed on the left, and proteins higher expressed by patients with a recurrence are displayed right. The protein fold change on a log_2_ scale is shown on the *x*-axis, with the significance indicated by the −log_10_ scale on the *y*-axis. The significance cutoff (*P* value = 0.05) is indicated with the black dotted line, showing significant increased proteins for patients without a recurrence in green and for patients with a recurrence in red. A two-tailed unpaired Welch *t* test was used to determine statistical significance between samples of patients with and without a recurrence.

Paired serum samples of 64 patients with stage III melanoma that progressed to stage IV melanoma were analyzed using DIA MS (Fig. 1; Fig. 2A). In this paired analysis, we observed that 70 proteins (out of 445 proteins with at least an observation in the majority of patients in both stage III and IV) were significantly higher expressed when patients developed stage IV disease (Fig. 2B). A large increase in CRP levels was observed in patients at time of stage IV disease compared with stage III disease (*P* < 0.0001; Fig. 2B; Supplementary Fig. S3A). In addition, the previously identified markers SAA1 (14) and LDHB (12) were also increased when stage III patients progressed to stage IV melanoma (Fig. 2B; Supplementary Fig. S3B and S3C).

To further evaluate differential expression of systemic cytokines and chemokines, we analyzed 33 paired stage III and stage IV patients for Olink proteomic assay (Supplementary Table S1; Fig. 1), because (usually) smaller and less abundant cytokines are difficult to detect by MS (Fig. 2A). This approach allowed us to evaluate 92 immuno-oncology markers. A significant higher expression of the cytokines IL8 (*P* = 0.0011) and IL10 (*P* = 0.0038) and adhesion G protein–coupled receptor G1 (ADGRG1) were observed in stage IV patients, whereas lower serum levels of inducible T-cell costimulatory ligand (ICOSLG; *P* = 0.0002) were detected (Supplementary Fig. S4A and S4B). To confirm these findings in an independent cohort, pretreatment protein expression of 86 stage III patients were compared with 22 stage IV patients. This unpaired analysis confirmed the increased expression of IL8 (*P* = 0.0080) and IL10 (*P* = 0.0228; Supplementary Fig. S4C and S4D) in patients with stage IV disease. These cytokines have also previously been associated with melanoma disease progression (23–26). In summary, these data show that upon disease progression increased systemic levels of CRP, SAA1, LDHB, IL8, and IL10 are observed upon disease progression in matched patient samples, validating previously found observations.

### Complement Factor B, Component 7, and Alpha-1B Glycoprotein Expression are Increased Upon Melanoma Progression and Recurrence

We next asked which markers that were increased with disease progression to stage IV disease overlapped with disease recurrence upon neoadjuvant therapy in stage III disease (Fig. 2C). Therefore, we next analyzed baseline plasma samples of patients with stage III melanoma that were treated with dual neoadjuvant ICB (OpACIN-neo study NCT02977052; Supplementary Table S2; refs. 7, 18). Patients who achieved a pathologic response seldom relapsed [2-year relapse-free survival (RFS) 97%], whereas those without a pathologic response had a poor RFS (2-year 36%; ref. 18). Therefore, it is particularly important to investigate predicting biomarkers for recurrent disease within the nonresponding patient cohort to identify patients that might require intensified therapies. Accordingly, we compared baseline plasma of patients without a pathologic response that had a recurrence to those without a recurrence (*n* = 21; Fig. 2C and D; Supplementary Fig. S1).

Comparing significant increased proteins of patients with stage IV melanoma that were shared with significantly increased proteins in baseline samples of nonresponding stage III patients with recurrent disease revealed an overlap for leucine-rich alpha-2-glycoprotein 1 (LRG1), alpha-1B glycoprotein (A1BG), and complement factor/component (Fig. 2B–D). The complement component (C7), complement factor B (CFB), and A1BG showed a significant increased expression in patients with stage IV melanoma (*P* = 0.0070, *P* = 0.0107, *P* = 0.0371, respectively) and nonresponding patients with a recurrence (*P* = 0.0302, *P* = 0.0400, *P* = 0.0208, respectively). However, no significant association was observed with pathologic response to neoadjuvant ICB for these markers (Supplementary Fig. S5A–S5C).

Next, we evaluated whether certain expression levels of C7, CFB, and A1BG could predict event-free survival (EFS) for patients without a response upon neoadjuvant ipilimumab and nivolumab. Optimal cutoffs for these markers were defined on the basis of summary ROC (sROC) curves, using the complete OpACIN-neo patient cohort (responding and nonresponding patients, *n* = 82 for C7; *n* = 83 for CFB and A1BG). We identified 23.2738, 26.212, and 27.2107, as the optimal cutoff for protein abundance of C7, CFB, and A1BG, respectively, resulting in an area under the sROC curve (AUC) of 0.621, 0.628, and 0.698, respectively. Patients with a high versus low C7 expression showed no significant difference in EFS (*P* = 0.062; Supplementary Fig. S5D), while patients with either high CFB or high A1BG expression had a significantly lower EFS following neoadjuvant ICB treatment (*P* = 0.016, *P* = 0.0064; Supplementary Fig. S5E and S5F).

To validate the prognostic impact of systemic expression of CFB and A1BG, baseline plasma samples of a second cohort of patients (*n* = 49) treated with neoadjuvant ipilimumab and nivolumab (8) were analyzed (Supplementary Table S3; Supplementary Fig. S2). The samples were analyzed on a different mass spectrometer, and therefore a new optimal cutoff was calculated. The AUC to discriminate between patients with and without a recurrence for CFB and A1BG was lower in the PRADO cohort compared with the OpACIN-neo cohort (CFB: 0.572, A1BG: 0.609). Moreover, no significant difference for EFS was observed for nonresponding patients with either a high CFB or A1BG compared with a low CFB or A1BG expression, respectively (Supplementary Fig. S5G and S5H). Thus, these markers could not be validated in an independent cohort, and therefore it remains uncertain whether systemic CFB and A1BG have prognostic potential for disease recurrence in nonresponding patients with stage III melanoma treated with neoadjuvant ICB.

### LRG1 Expression is Associated with Melanoma Progression and Recurrence

**FIGURE 3:**
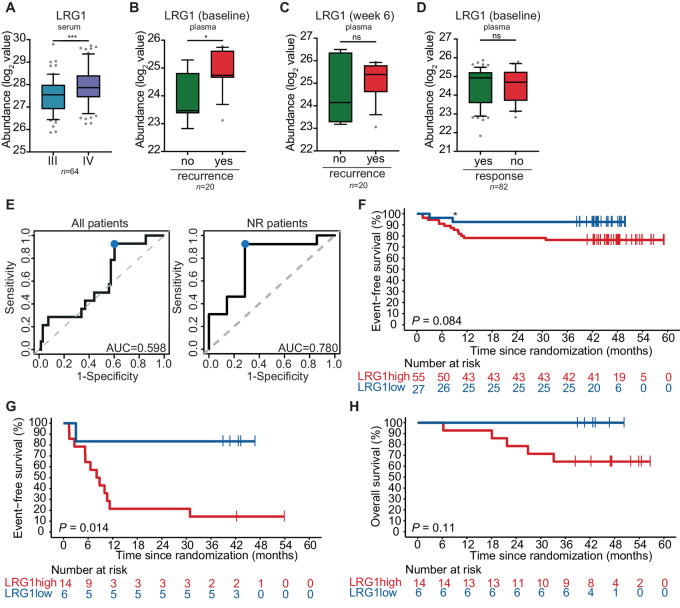
LRG1 expression is associated with melanoma progression and recurrence. **A–D,** Normalized protein expression (log_2_) values of LRG1, measured by MS for matched stage III and IV patients (*n* = 64; **A**), non-responsive patients at baseline of the OpACIN-neo study (*n* = 20; **B**), and at week 6 after neoadjuvant ICB treatment of the OpACIN-neo study (*n* = 20; **C**) and all patients at baseline of the OpACIN-neo study (*n* = 82; **D**). A two-tailed paired Student *t* test was used to determine statistical significance between stage III and stage IV samples. A two-tailed unpaired Student *t* test was used to compare patients with and without a recurrence or response of the OpACIN-neo study. **E,** sROC curves to define the optimal cutoff (marked by the blue dot) for all patients (left) and nonresponding patients (right) of the OpACIN-neo study for baseline LRG1 expression. The AUC for all patients was 0.598 and for nonresponder patients 0.780, with an optimal cutoff of 24.5504. A Kaplan–Meier curve showing EFS for all patients (**F**) and nonresponder patients (**G**) of the OpACIN-neo study with either a high (red) or low (blue) expression of LRG1. The asterisk denotes a patient (in LRG1 low group) who died because of irAEs. **H,** A Kaplan–Meier curve showing overall survival for nonresponder patients of the OpACIN-neo study with either a high (red) or low (blue) expression of LRG1. *P* value was calculated using the log-rank test (two-sided) and significance is indicated. *, *P* < 0.05; ***, *P* <0.001.

The analysis of significantly increased proteins associated with both disease progression and disease recurrence also identified LRG1 as an overlapping marker (Fig. 2B–D). A significantly higher expression of LRG1 was observed in serum of stage IV disease patients compared with the matching stage III samples (*P* = 0.0002; Fig. 3A). For nonresponding patients of the OpACIN-neo cohort, there was a significantly higher expression of LRG1 in baseline samples for patients with a recurrence in comparison with patients without a recurrence (*P* = 0.0156; Fig. 3B). This significant difference was only significant at baseline, and no significant difference was found after neoadjuvant ICB at week 6 (moment of surgery; Fig. 3C). When evaluating the prognostic value of LRG1 for pathologic response, there was no significant difference between responding and nonresponding patients upon neoadjuvant ICB treatment (Fig. 3D).

Subsequently, we assessed whether we were able to predict at baseline if OpACIN-neo patients were more likely to have a recurrence based on their LRG1 expression, identifying 24.5504 as the optimal cutoff for LRG1 based on the sROC curve. The AUC was higher for nonresponding patients (0.780) compared with the whole patient cohort (0.598; Fig. 3E). On the basis of this cutoff, we could discriminate patients with a high and low baseline LRG1 protein expression. When comparing both groups in the total cohort of all responding and nonresponding patients, no significant difference in EFS was found (*P* = 0.084; Fig. 3F). However, when patients with a high versus low baseline LRG1 expression were compared in the subgroup of patients that had no response upon neoadjuvant ICB treatment, a significantly lower EFS was observed in patients with a high LRG1 expression (*P* = 0.014; Fig. 3G). After a median follow-up of 47 months, 5 patients with high LRG1 expression have died, while none of the patients with low LRG1 expression has died. This difference was not (yet) significant (*P* = 0.11; Fig. 3H).

As neutrophils are among the cell types that secrete LRG1 (27), we analyzed whether baseline neutrophil count was associated with systemic LRG1 expression. No correlation between systemic LRG1 expression and neutrophil count was observed (Supplementary Fig. S6A). Furthermore, high neutrophil count and high neutrophil-to-lymphocyte ratio, which has been associated with poor prognosis (28), was not significantly associated with recurrence in nonresponding patients (Supplementary Fig. S6B and S6C).

### LRG1 is Associated with Melanoma Recurrence in a Second Independent Cohort of Patients with Stage III Melanoma Treated with Neoadjuvant Ipilimumab + Nivolumab

**FIGURE 4:**
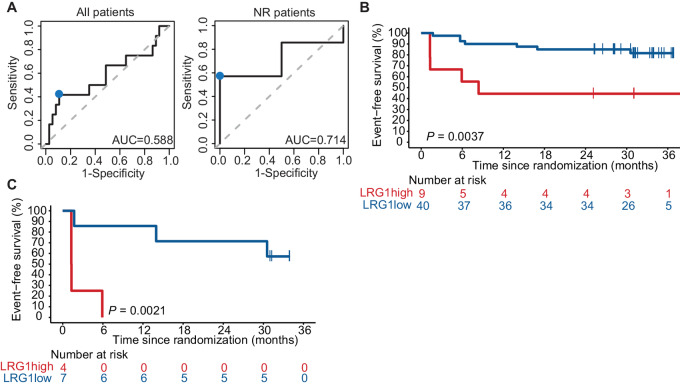
LRG1 expression is associated with melanoma recurrence in a second independent cohort. **A,** sROC curves to define the optimal cutoff (marked by the blue dot) for all patients (left) and nonresponding patients (right) of the PRADO study for baseline LRG1 expression in patient plasma. The AUC for all patients was 0.588 and for nonresponder patients 0.714, with an optimal cutoff of 24.9485. A Kaplan–Meier curve showing EFS for all patients (*n* = 49; **B**) and nonresponder patients (*n* = 11; **C**) of the PRADO study with either a high (red) or low (blue) expression LRG1. *P* value was calculated using the log-rank test (two-sided) and significance is indicated.

To confirm the prognostic potential of LRG1, we analyzed pretreatment plasma samples of a second independent cohort (PRADO) of patients who were also treated with neoadjuvant ipilimumab and nivolumab (Supplementary Table S3; Supplementary Fig. S2; ref. 8). In this cohort, we determined whether we were also able to identify patients who had a recurrence based on their LRG1 expression, using the sROC curves to define the optimal cutoff. A different cutoff was used, because this cohort was analyzed on a different mass spectrometer. Using this strategy, a cutoff for LRG1 of 24.9485 was determined, corresponding to an AUC of 0.588 for all patients and 0.714 for patients not responding to treatment (Fig. 4A). Consequently, patients were divided into LRG1-high and LRG1-low baseline protein expression. Patients with a high LRG1 expression had a significantly lower 2-year EFS compared with patients with a low LRG1 expression (*P* = 0.0037; Fig. 4B). This was even more pronounced in the nonresponding patients, where all patients with a high LRG1 expression experienced a disease recurrence within 6 months after start treatment versus only 14% in the LRG1 low group (*P* = 0.0021; Fig. 4C).

In summary, these data validate in two independent cohorts that pretreatment systemic LRG1 expression is a prognostic marker for disease recurrence after neoadjuvant treatment with ICB, especially in patients who do not respond to neoadjuvant ICB.

### High LRG1 Expression is Associated with Distant Metastasis in Patients That do not Respond to Neoadjuvant Ipilimumab and Nivolumab

**FIGURE 5:**
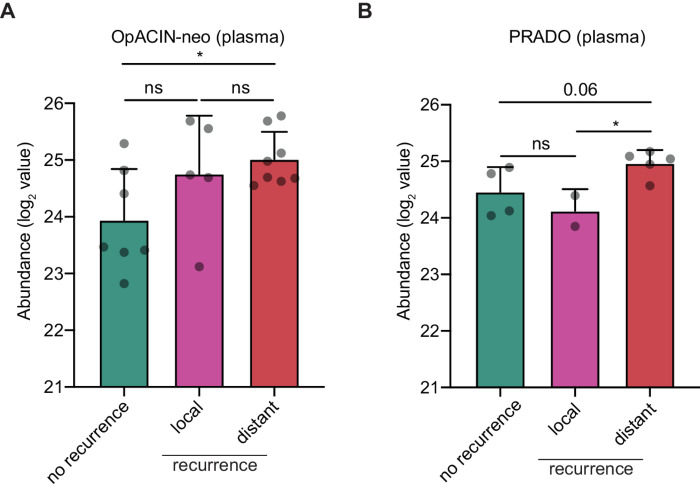
High LRG1 expression is associated with distant metastasis in patients that do not respond to neoadjuvant ipilimumab and nivolumab. LRG1 expression measured in patient plasma by MS for patients without a response upon neoadjuvant ICB of the OpACIN-neo study (*n* = 20; **A**) and PRADO study (*n* = 11; **B**). Comparing patients without a recurrence (green), patients with a local recurrence (pink) and patients with a distant recurrence (red). A two-tailed unpaired Student *t* test for comparing patient groups. *, *P* < 0.05.

Patients without a pathologic response after neoadjuvant ICB therapy were more likely to develop a recurrence compared with patients with a pathologic response (8, 18, 29). These recurrences were either at local or distant sites. Next, we assessed whether LRG1 was associated with the site of recurrence. We observed that all nonresponder patients that developed a distant metastasis showed higher baseline systemic LRG1 protein expression. In the OpACIN-neo study, a significantly higher expression of LRG1 was found at baseline for patients that had a distant recurrence compared with patients without recurrent disease (*P* = 0.0159), whereas no significant difference was found compared with local recurrence (Fig. 5A). Comparing LRG1 expression of patients that developed a distant recurrence to patients without a recurrence in PRADO, only a trend for increased levels of LRG1 was found (*P* = 0.0618; Fig. 5B). This analysis showed that patients that developed a distant recurrence have a higher pretreatment expression of LRG1 compared with patients without a recurrence or those that recurred at a local site only.

## Discussion

Treatment of patients with advanced-stage melanoma with ICB has demonstrated unprecedented success (30, 31). In the setting of neoadjuvant treatment even higher response rates are achieved (4, 7, 8, 30, 32). In our cohort (OpACIN-neo and PRADO study NCT02977052), high pathologic response rates, with durable cancer control were observed with 2-year RFS of 84%–85%. Still, a subset of patients did not respond (24%–28% of patients) and these patients were much more likely to develop a recurrence of melanoma. Additional adjuvant treatment of nonresponding patients after neoadjuvant ICB increased the 2-year RFS from 36% to 71% (8, 18), highlighting that this patient group could benefit from additional therapies. Hence, it is particularly important to identify patients without a pathologic response after neoadjuvant ICB who are at risk for disease recurrence and therefore could benefit from adjuvant therapy. On the basis of the observation that systemic immune response is required for immunotherapeutic efficacy (9, 33), we postulated that patients with disease progression and recurrence have a higher degree of systemic immune suppression, hampering an effective immune response. Accordingly, we analyzed the systemic protein expression of patients with melanoma with MS-based protein profiling and Olink analysis using an unbiased approach.

In our screen, we confirmed previously identified markers CRP, SAA1, LDHB, IL8, and IL10 (12–14, 23–25) to be associated with disease progression, making our patient cohort for disease progression a representative cohort. Of note, IL8 and IL10 were not detected by MS, because most cytokines are difficult to identify by MS, which prompted us to conduct Olink analysis. The cohort included matched patients that progressed from stage III to stage IV melanoma, making this a unique patient group.

Our study also has some limitations that need to be considered. All the analyses were performed without a correction for multiple testing. In addition, all patients progressed to stage IV disease, thus there is a selection bias for patients with worse prognosis. Another limitation of our cohort is the low number of patients, especially the number of nonresponding patients upon neoadjuvant therapy in OpACIN-neo and PRADO cohort is small. Moreover, not all markers were validated in the different independent cohorts and different cutoff for these markers were used, because the patient samples of the OpACIN-neo and PRADO cohort were measured on different mass spectrometers. Furthermore, nonresponding patients in the PRADO cohort received adjuvant therapy, while this was not applied in the OpACIN-neo cohort. Future studies that use a uniform cutoff and includes statistical adjustment of the data for multiple testing for a higher number of patients will strengthen the findings of this study. Nevertheless, we consider these homogeneous cohorts as the best available to identify circulating proteins that are potentially associated with systemic immune suppression. In particular, comparing the results from these patients with progressive disease to two independent cohorts of patients with stage III melanoma treated with neoadjuvant treatment, with only differences in dose levels in the treatment regimes, allowed us to more confidently identify markers associated with disease progression as well as disease recurrence.

In addition, when comparing proteins between patients with stage IV with progressive disease and initial stage III patients with recurrent local or distant disease, increased LRG1 expression was found in both patient cohorts. LRG1 is a secreted glycoprotein that is constitutively synthesized by hepatocytes and neutrophils under physiological conditions (27). Following various inflammatory stimuli, including IL1β, IL6, IL10, IL17, IL22, IL33, TNFα and TGFβ, secretion of LRG1 is increased predominantly by hepatocytes, neutrophils and endothelial cells and can be detected systemically and/or at the local tissue level (34). Tumor and stromal cells within the tumor microenvironment (TME) can also be a source of LRG1, and circulating levels of LRG1 were previously shown to be correlated with disease progression, disease burden, and poor prognosis in different cancer types (e.g., gastrointestinal, lung, pancreatic, prostate cancer; refs. 35–41), but not melanoma.

Here, we show for the first time that systemic LRG1 expression is also increased during melanoma disease progression. Moreover, pretreatment elevated circulating levels of LRG1 were also associated with poor patient outcome after neoadjuvant ICB therapy. These data further support the relevance of LRG1 in cancer (progression).

LRG1 has previously been described to promote cancer pathogenesis, either directly or indirectly. LRG1 contributes directly to tumor cell viability and proliferation (42, 43), promoting epithelial-to-mesenchymal transition (34) and promoting dysfunctional angiogenesis (44). It also acts indirectly by modifying the TGFβ signaling pathway (45) and enhancing expression of proangiogenic factors (VEGFA and angiopoietin-1; refs. 46, 47). TGFβ signaling has been shown to directly inhibit antitumor immune responses (48).

This is in line with our observation that LRG1 is associated with disease recurrence, and nonresponding patients had a particularly poor prognosis when increased LRG1 expression was observed. Furthermore, the highest expression of LRG1 was found in patients that developed a distant metastasis. Our findings support previous findings that LRG1 enhances metastatic dissemination and contributes to the metastatic niche (34). However, further investigations are required to determine whether LRG1 is indeed mechanistically contributing to metastasis formation in patients with melanoma.

Factors from the complement system, CFB and C7, and A1BG were also found to be significantly increased in both patients with progressive and recurrent disease. However, in our independent cohort, these findings could not be confirmed. A1BG, a member of the immunoglobulin superfamily, with unknown function (49), has been described to be elevated in pancreatic ductal adenocarcinoma (50) and urinary samples from bladder cancer patients (51). The complement system plays a major, but complex role in cancer due to opposing effects, which is dependent of the context (site activation, composition TME, tumor cell sensitivity to complement; ref. 52). Although previous studies support a negative role for CFB (53) in squamous cell carcinoma mouse models, and C7 in patients with glioma (54), the role of these complement factors in melanoma disease recurrence needs to be further elucidated.

Biomarkers that are associated with disease recurrence and progression could serve as therapeutic targets, especially when these markers are causal to immune suppressive effects. Because IL8 was previously shown to be expressed in higher levels in patients that progressed from II to stage III melanoma (23), it is a poor prognostic marker in stage IV melanoma, and a decrease in levels from baseline are correlated with response to anti-PD-1 treatment (25), an anti-IL8 antibody (HuMax-IL8, BMS-986253) has been developed. In a study (NCT03400332) testing nivolumab + anti-IL8 therapy in patients with increased IL8 serum levels showed dose-proportional pharmacokinetics and reduction in serum IL8 levels, resulting in partial responses (55).

Considering our data that show increased levels of LRG1 to be associated with disease progression and recurrence, novel treatment strategies could explore the therapeutic potential of targeting LRG1. Currently, anti-LRG1 treatment has only been tested in mouse models, and showed reduced tumor growth and synergistic effect with anti-PD-1 (44, 56). In addition, anti-LRG1 improved vascular function, and therefore, it is hypothesized that this vascular normalization leads to improved delivery of immunotherapies (or other therapies; refs. 44, 57). Moreover, it has been shown that systemic immune changes can be reversed, revealing the plasticity of the systemic immune state (33). Although these preclinical results are promising, further studies are needed to assess the clinical therapeutic utility of anti-LRG1 therapy. In addition, it would be of interest to assess whether LRG1 levels are elevated in nonresponding patients after surgery when adjuvant treatment decisions are made.

In conclusion, we identified LRG1 as a potential biomarker for recurrence in patients treated with neoadjuvant ICB, which could serve as marker for intensified adjuvant treatment and follow-up. Given the cumulating data on LRG-1 and cancer progression, further supported by mouse data indicating improved tumor control upon LRG-1 inhibition in combination with ICB, we envision that LRG1 could become, not only a biomarker, but also a possible target for combination therapy with ICB for patients with an unfavorable response after treatment with neoadjuvant immunotherapy.

## Supplementary Material

Table S1 Table S1 shows an overview evaluated immuno-oncology markers by Olink proteomic assay

Table S2 Table S2 shows the patient characteristics OpACIN-neo

Table S3 Table S3 shows the patient characteristics PRADO

Figure S1 Figure S1 shows a flowchart of patients OpACIN-neo study

Figure S2 Figure S2 shows a flowchart of patients PRADO study

Figure S3 Figure S3 shows that SAA1, CRP and LDHB are associated with melanoma disease progression.

Figure S4 Figure S4 shows the serum analysis using Olink proteomic assay

Figure S5 Figure S5 shows that complement factor B (CFB), component 7 (C7) and alpha-1B-glycoportein (A1BG) expression are increased upon melanoma progression and recurrence.

Figure S6 Figure S6 shows that neutrophil count and neutrophil-to-lymphocyte ratio (NLR) are not associated with recurrence in non-responding patients
