# Supplementary material for: Systemic LRG1 Expression in Melanoma is Associated with Disease Progression and Recurrence
Source: Cancer Res Commun. 2023 Apr 20;3(4):672–83. doi: 10.1158/2767-9764.CRC-23-0015 (PMC10117404; doi:10.1158/2767-9764.CRC-23-0015)
Supplement: Table S1 — shows an overview evaluated immuno-oncology markers by Olink proteomic assay [file crc-23-0015-s01.pdf]

Table S1: Overview evaluated immuno-oncology markers by Olink proteomic assay

Immuno-oncology markers							
IL-8	TNFRSF9	TIE2	MCP-3	CD40-L	IL-1α	CD244	EGF
PGF	IL-6	ADGRG1	MCP-1	CRTAM	CXCL11	MCP-4	TRAIL
FGF2	CXCL9	CD8A	CAIX	IFN-β	ADA	CD4	NOS3
IL-2	Gal-9	VEGFR-2	CD40	IL-18	GZMH	VEGFC	IL-12
CXCL1	TNFSF14	IL-33	TWEAK	CSF-1	PDCD1	FASLG	CD28
CCL19	MCP-2	CCL4	IL-35	Gal-1	PD-L1	CD27	CXCL5
IL-5	HGF	GZMA	HO-1	CX3CL1	CXCL10	CD70	IL-10
CD83	CCL23	CD5	CCL3	MMP7	ARG1	NCR1	DCN
TNFRSF21	TNFRSF4	MIC-A/B	CCL17	ANGPT2	PTN	CXCL12	IFN-γ
LAMP3	CASP-8	ICOSLG	MMP12	CXCL13	PD-L2	VEGFA	IL-4
IL-21	IL12RB1	IL-13	CCL20	TNF	KLRD1	GZMB	TNFRSF12A
PDGF subunit B	LAP TGF-β-1						
