# Supplementary material for: Systemic LRG1 Expression in Melanoma is Associated with Disease Progression and Recurrence
Source: Cancer Res Commun. 2023 Apr 20;3(4):672–83. doi: 10.1158/2767-9764.CRC-23-0015 (PMC10117404; doi:10.1158/2767-9764.CRC-23-0015)
Supplement: Table S2 — shows the patient characteristics OpACIN-neo [file crc-23-0015-s02.pdf]

Table S2: Patient characteristics OpACIN-neo

Characteristics	Total cohort (n=86)	
Institute		
Netherlands Cancer Institute	46	(53%)
Melanoma Institute Australia	38	(44%)
Karolinska Institute	2	(2%)
Age, years (median, IQR)	58, 45-65	
Sex		
Men	49	(57%)
Women	37	(43%)
Primary tumor stage		
T1a/b	29	(34%)
T2a/b	9	(10%)
T3a/b	14	(16%)
T4a/b	10	(12%)
Tx	24	(28%)
Treatment		
2x IPI 3 mg kg^{-1} + NIVO 1 mg kg^{-1} q3wk	30	(35%)
2x IPI 1 mg kg^{-1} + NIVO 3 mg kg^{-1} q3wk	30	(35%)
2x IPI 3 mg kg^{-1} q3wk directly followed by 2x NIVO 3 mg kg^{-1} q3wk	26	(30%)
Pathologic response		
Yes	64	(74%)
No	21	(24%)
NE[a]	1	(1%)
Recurrence[b]		
Yes	16	(19%)
No	70	(81%)
Alive[b]		
Yes	79	(92%)
No	7	(8%)

Percentages may not sum up to 100 because of rounding.

[a] Patient did not undergo surgery because of toxicity

[b] At data cutoff (14 February 2022), the median follow-up from data of registration was 47 months, with a minimum follow-up of 38 months for all patients alive.

IPI; ipilimumab, NIVO; nivolumab, IQR; interquartile range
