# Supplementary material for: Systemic LRG1 Expression in Melanoma is Associated with Disease Progression and Recurrence
Source: Cancer Res Commun. 2023 Apr 20;3(4):672–83. doi: 10.1158/2767-9764.CRC-23-0015 (PMC10117404; doi:10.1158/2767-9764.CRC-23-0015)
Supplement: Table S3 — shows the patient characteristics PRADO [file crc-23-0015-s03.pdf]

Table S3: Patient characteristics PRADO

Characteristics	Total cohort (n=49)	
Institute		
Netherlands Cancer Institute	49	(100%)
Age, years (median, IQR)	58, 52-68	
Sex		
Men	32	(65%)
Women	17	(35%)
Primary tumor stage		
T1a/b	6	(12%)
T2a/b	13	(27%)
T3a/b	13	(27%)
T4a/b	12	(24%)
Tx	1	(2%)
Unknown primary	4	(8%)
Pathologic response		
Yes	38	(78%)
No	11	(22%)
Recurrence[a]		
Yes	12	(24%)
No	37	(76%)
Alive[a]		
Yes	45	(92%)
No	4	(8%)

[a] At data cutoff (7 February 2022), the median follow-up from data of registration was 28.1 months, with a minimum follow-up of 23.4 months for all patients alive.

IQR; interquartile range
