# Supplementary material for: Systemic LRG1 Expression in Melanoma is Associated with Disease Progression and Recurrence
Source: Cancer Res Commun. 2023 Apr 20;3(4):672–83. doi: 10.1158/2767-9764.CRC-23-0015 (PMC10117404; doi:10.1158/2767-9764.CRC-23-0015)
Supplement: Figure S1 — shows a flowchart of patients OpACIN-neo study [file crc-23-0015-s04.pdf]

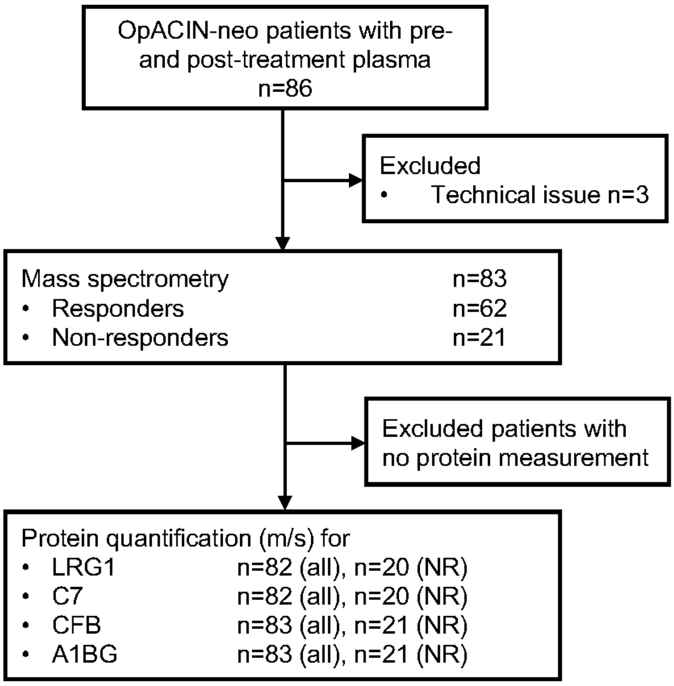

Figure S1: Flowchart of patients OpACIN-neo study. Number of patients included for mass spectrometry. For every patient, two plasma samples were analyzed: one baseline samples (pre-treatment) sample and one post-treatment (after neoadjuvant ICB therapy) sample at week 6. Mass spectrometry data was available for 83 patients that had measurements for both pre-treatment and post-treatment samples. Patients with no measurement for certain proteins (e.g. LRG1 and C7) were excluded from the corresponding analysis. NR; non-responding patients
