# Supplementary material for: Systemic LRG1 Expression in Melanoma is Associated with Disease Progression and Recurrence
Source: Cancer Res Commun. 2023 Apr 20;3(4):672–83. doi: 10.1158/2767-9764.CRC-23-0015 (PMC10117404; doi:10.1158/2767-9764.CRC-23-0015)
Supplement: Figure S2 — shows a flowchart of patients PRADO study [file crc-23-0015-s05.pdf]

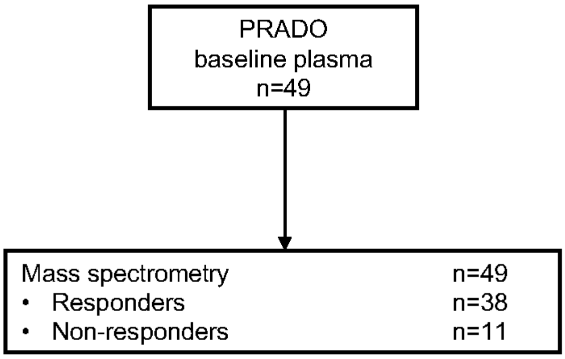

Figure S2: Flowchart of patients PRADO study. A total of 49 patients from the PRADO study were included for mass spectrometry. The baseline (pre-treatment) plasma samples of patients were analyzed.
