# Supplementary material for: Systemic LRG1 Expression in Melanoma is Associated with Disease Progression and Recurrence
Source: Cancer Res Commun. 2023 Apr 20;3(4):672–83. doi: 10.1158/2767-9764.CRC-23-0015 (PMC10117404; doi:10.1158/2767-9764.CRC-23-0015)
Supplement: Figure S3 — shows that SAA1, CRP and LDHB are associated with melanoma disease progression. [file crc-23-0015-s06.pdf]

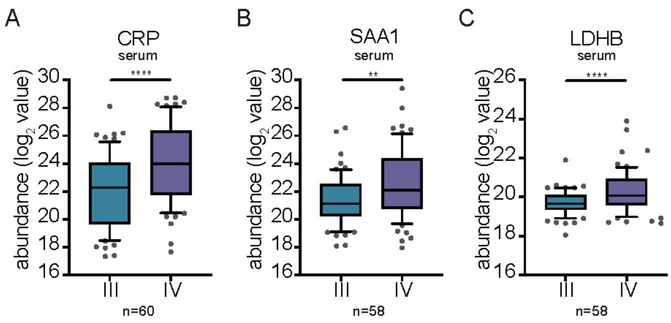

Figure S3: SAA1, CRP and LDHB are associated with melanoma disease progression. Serum expression of **(A)** C-reactive protein (CRP) (n=60), **(B)** serum amyloid A1 (SAA1) (n=58) and **(C)** lactate dehydrogenase B (LDHB) (n=58) by patients with stage III disease that developed stage IV disease, as measured by mass spectrometry. A two-tailed paired Student's *t*-test was used to determine statistical significance between stage III and stage IV samples. **, *P* <0.01, ****, *P* <0.0001
