# Supplementary material for: Systemic LRG1 Expression in Melanoma is Associated with Disease Progression and Recurrence
Source: Cancer Res Commun. 2023 Apr 20;3(4):672–83. doi: 10.1158/2767-9764.CRC-23-0015 (PMC10117404; doi:10.1158/2767-9764.CRC-23-0015)
Supplement: Figure S4 — shows the serum analysis using Olink proteomic assay [file crc-23-0015-s07.pdf]

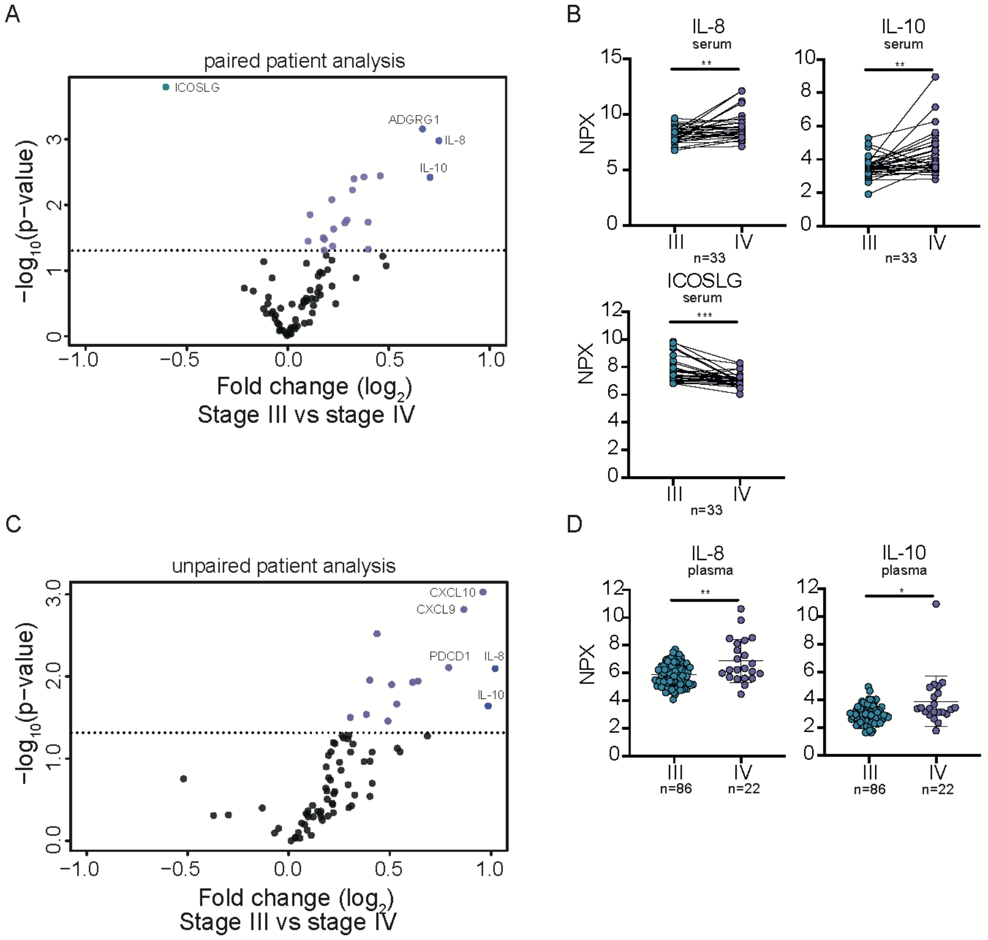

Figure S4: Serum analysis using Olink proteomic assay. (A) Volcano plot showing differential marker expression of serum analysis using Olink immunoassay, comparing protein expression between matched patients with stage III and stage IV disease (n=33). Proteins with higher expression at stage III disease are displayed on the left, and proteins highly expressed at stage IV are displayed on the right. The protein fold change on a log_2 scale is shown on the x-axis, with the significance indicated by the $-log_{10}$ scale on the y-axis. The significance cut-off (*p*-value=0.05) is indicated with the black dotted line, showing significant increased proteins for stage III in blue and for stage IV in purple. A two-tailed paired Student's *t*-test was used to determine statistical significance between stage III and stage IV samples. **(B)** Normalized protein expression of significant proteins of (A) at stage III and stage IV for paired patients (n=33). Significance was assessed by a paired Student's *t*-test. **(C)** Volcano plot showing differential marker expression of plasma analysis using Olink immunoassay, comparing protein expression between patients with stage III (n=22) and stage IV (n=86) disease (not matched). Proteins higher expressed at stage III disease are displayed on the left, and proteins higher expressed at stage IV are displayed on the right. The protein fold change on a log_2 scale is shown on the x-axis, with the significance indicated by the $-log_{10}$ scale on the y-axis. The significance cut-off (*p*-value=0.05) is indicated with the black dotted line, showing significant increased proteins for stage IV in purple. A two-tailed unpaired Welch's *t*-test was used to determine statistical significance between stage III and stage IV samples. **(D)** Normalized protein expression for IL-8 and IL-10. Significance was assessed by an unpaired Welch's *t*-test between stage III and stage IV patients. *, $P<0.05$, **, $P<0.01$, ***, $P<0.001$
