# Supplementary material for: Systemic LRG1 Expression in Melanoma is Associated with Disease Progression and Recurrence
Source: Cancer Res Commun. 2023 Apr 20;3(4):672–83. doi: 10.1158/2767-9764.CRC-23-0015 (PMC10117404; doi:10.1158/2767-9764.CRC-23-0015)
Supplement: Figure S5 — shows that complement factor B (CFB), component 7 (C7) and alpha-1B-glycoportein (A1BG) expression are increased upon melanoma progression and recurrence. [file crc-23-0015-s08.pdf]

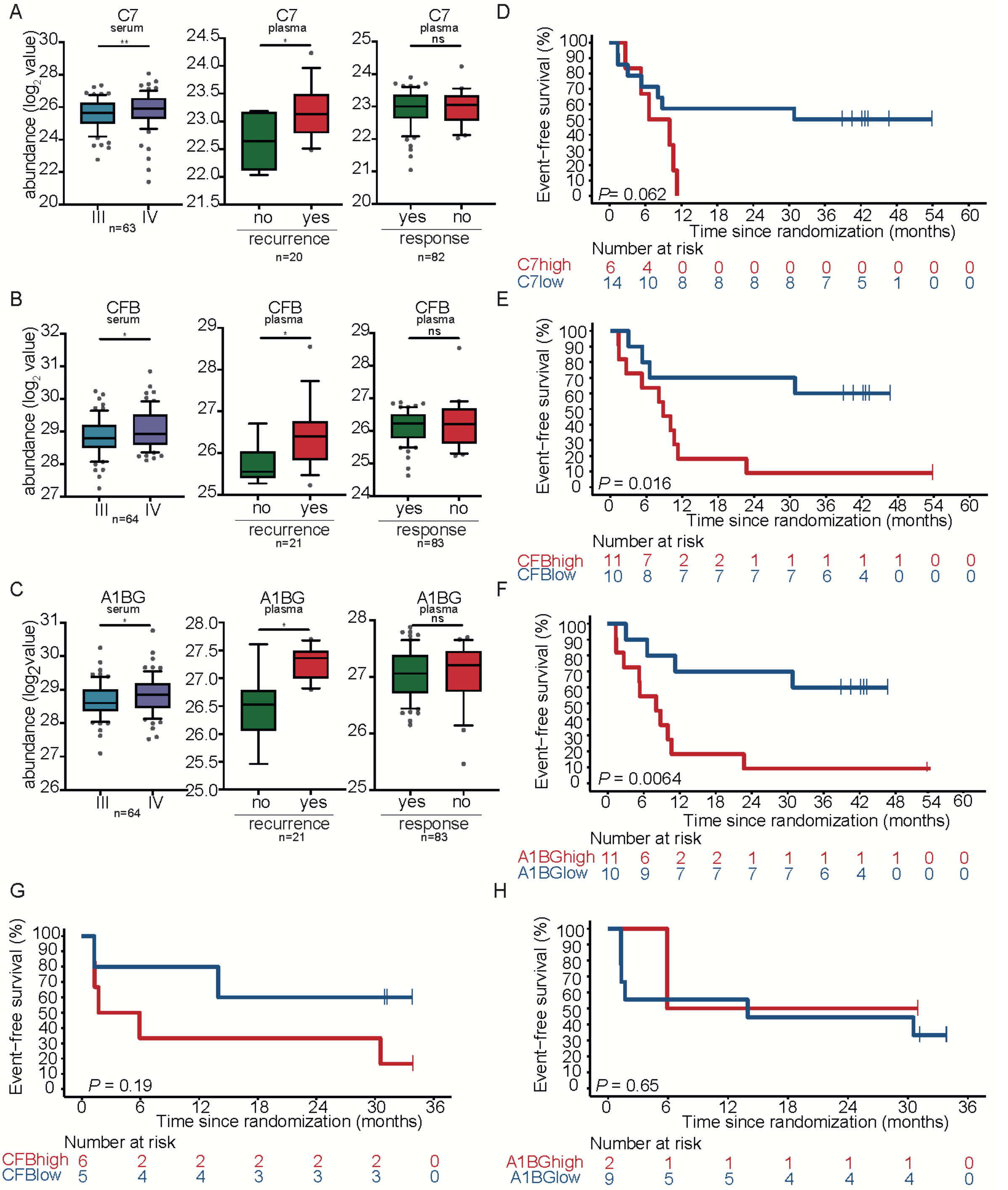

Figure S5: Complement factor B (CFB), component 7 (C7) expression and alpha-1B-glycoprotein (A1BG) are increased upon melanoma progression and recurrence. (A-C) Normalized protein expression ($\log_2$) values of **(A)** C7, **(B)** CFB and **(C)** A1BG, measured by mass spectrometry for matched stage III and IV patients (n=63 for C7, n=64 for CFB and A1BG), non-responsive patients of OpACIN-neo study (n=20 for C7, n=21 for CFB and A1BG) and all patients of the OpACIN-neo study (n=82 for C7, n=83 for CFB and A1BG). A two-tailed paired Student's *t*-test was used to determine statistical significance between stage III and stage IV samples. A two-tailed unpaired Student's *t*-test for comparing patients with and without a recurrence or response of the OpACIN-neo study, using a Welch's *t*-test when variances were significantly different. **(D-F)** A Kaplan Meier curve showing event-free survival (EFS) for non-responder patients of the OpACIN-neo study with either a high (red) or low (blue) expression of **(D)** C7, **(E)** CFB and **(F)** A1BG. *P* value was calculated using the log-rank test (two-sided) and significance is indicated. **(G-H)** A Kaplan Meier curve showing event-free survival (EFS) for non-responder patients

of the PRADO study with either a high (red) or low (blue) expression of **(G)** CFB and **(H)** A1BG. *P* value was calculated using the log-rank test (two-sided) and significance is indicated. *, $P<0.05$, **, $P<0.01$
