# Supplementary material for: Systemic LRG1 Expression in Melanoma is Associated with Disease Progression and Recurrence
Source: Cancer Res Commun. 2023 Apr 20;3(4):672–83. doi: 10.1158/2767-9764.CRC-23-0015 (PMC10117404; doi:10.1158/2767-9764.CRC-23-0015)
Supplement: Figure S6 — shows that neutrophil count and neutrophil-to-lymphocyte ratio (NLR) are not associated with recurrence in non-responding patients [file crc-23-0015-s09.pdf]

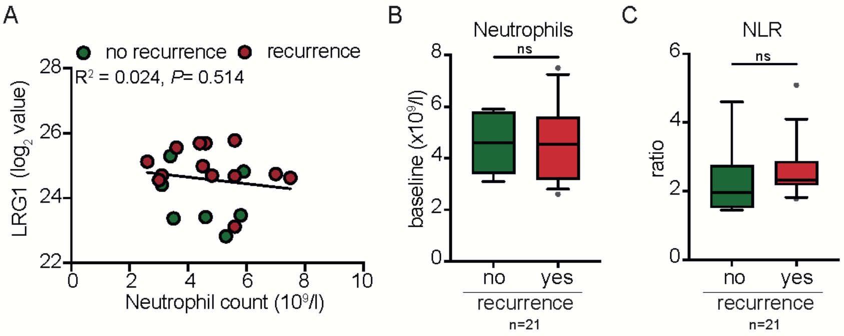

Figure S6: Neutrophil count and neutrophil-to-lymphocyte ratio (NLR) are not associated with recurrence in non-responding patients. Analysis of pre-treatment samples of non-responding patients of the OpACIN-neo study. **(A)** Correlation between systemic normalized LRG1 expression and neutrophil count for patients without a recurrence (green dots) or without a recurrence (red dots). The correlation coefficient and *p*-value were computed using the Pearson correlation method. **(B)** Neutrophil count and **(C)** neutrophil-to-lymphocyte ratio at baseline for non-responding patients with or without a response (n=21). A two-tailed unpaired Student's *t*-test was used to compare patients with and without a recurrence.
